# The COVID-19 Vaccination and IgA Nephropathy: Further Cause for Reassurance

**DOI:** 10.34067/KID.0000000000000530

**Published:** 2024-09-26

**Authors:** Haresh Selvaskandan, Jonathan Barratt

**Affiliations:** John Walls Renal Unit, University Hospitals of Leicester NHS Trust, Leicester, United Kingdom and Mayer IgA Nephropathy Laboratories, Department of Cardiovascular Sciences, University of Leicester, Leicester, United Kingdom

**Keywords:** COVID-19, IgA nephropathy

The coronavirus disease 2019 (COVID-19) pandemic caused unprecedented global disruption in 2020, leading to significant morbidity and mortality.^1^ Global lockdowns were instigated to contain the virus, which in turn had negative socioeconomic ramifications and indirectly contributed to further morbidity and mortality.^2^ The introduction of the COVID-19 vaccine altered the course of the pandemic. Its development, evaluation, approval, and subsequent global deployment was achieved in a remarkably short time frame, serving as an exemplar for internationally streamlined public health responses.

The vaccine muted the virulence of COVID-19,^3^ allowing lockdowns to ease and permitting those most at risk of severe disease to stop isolating, including those with kidney disease who were immunosuppressed. Although the benefits of the COVID-19 vaccine are now clear at a population level, it is also now established that a small proportion of recipients experience severe side effects, including myocarditis, cerebral venous thromboses, and even *de novo* kidney disease, possibly including IgA nephropathy.^4–6^ Although a cluster of cases in the literature report new diagnoses of IgA nephropathy after COVID-19 vaccination, compelling evidence indicating a true causal link between the vaccination and IgA nephropathy (over exacerbation of pre-existing but undiagnosed disease) is lacking. Several case reports/series describe flares of pre-existing IgA nephropathy after vaccination; however, the long-term effects of these flares are yet to be defined. In this issue, Aoki *et al.* attempt to address some of these concerns.^7^

Aoki *et al.* identified 127 patients who presented with visible hematuria across 22 centers in Japan, mostly within 3 days of receiving a COVID-19 vaccine. In 35 patients, a diagnosis of IgA nephropathy had already been made, with the vaccine producing a flare, resulting in visible hematuria. A kidney biopsy was performed in 70 of the 90 incident patients, and all were found to have histological features consistent with IgA nephropathy. Intriguingly, 64 of the 90 patients were already known to have pre-existing hematuria and/or proteinuria, with nine reporting episodes of visible hematuria before vaccination. Historical clinical data were not reported for the remaining 26 patients, so it is not clear if these patients did not have hematuria/proteinuria or were simply not tested. These findings indicate that at least in a Japanese cohort, the COVID-19 vaccine most often triggers a flare of pre-existing IgA nephropathy, and very rarely induces *de novo* IgA nephropathy, if at all. Flares (defined as visible hematuria in the context of IgA nephropathy) were associated with a transient rise in proteinuria, which settled at 6 months (reported at a cohort level), and did not appear to associate with a decline in kidney function over the same time frame.

The work by Aoki *et al.* provides further reassurance, with some caveats, to those with IgA nephropathy who may be apprehensive about receiving a COVID-19 vaccine. Although the prevalence of IgA nephropathy across the 22 participating centers was not reported, the overall incidence of flares after vaccination seems to be low. Among those who do experience a flare, any exacerbation in disease activity seems to be short-lived, with proteinuria surges settling after 6 months to a urine protein:creatinine ratio of around 0.3 g/g.

Further baseline and follow-up data from this cohort would be welcomed. Pre-vaccination proteinuria levels were not reported, and so it is unclear whether the proteinuria levels reported at 6 months were higher, lower, or at pre-vaccine baseline levels. Proteinuria levels 3 months before vaccination may allow for change in proteinuria to be calculated over a 9-month period, mirroring the surrogate end point for kidney failure used in many contemporary IgA nephropathy clinical trials. Similarly, the evaluation of serum creatinine over 6 months is likely to be too short a time frame to detect any meaningful changes in a disease that typically progresses over decades—collecting serum creatinine at 9 and 24 months and reporting eGFR slopes at these time points may again mirror surrogate end points used in clinical trials and provide further reassurance.

Aoki *et al.* offered a cohort of 78 patients diagnosed with IgA nephropathy before the pandemic as a control (non-vaccinated group) against which the characteristics of those newly diagnosed after COVID-19 vaccination were compared (vaccinated group). They found those in the vaccinated group had lower rates of tubular atrophy/fibrosis (T1/2) and endocapillary hypercellularity (E1). While the lower incidence of T1/T2 may be attributable to lead-time bias with regards to diagnosis (vaccination triggered visible hematuria prompted an earlier diagnosis of otherwise unrecognized IgA nephropathy), the latter observation may offer insights into the mechanisms by which COVID-19 vaccinations induce IgA nephropathy flares. Accumulating evidence supports a role for a mucosal–kidney axis in IgA nephropathy,^8^ and studies which investigated mucosal versus systemic antigenic stimuli among patients with IgA nephropathy and healthy participants found increased production of poorly*-O-*galactosylated IgA1 (gd-IgA1) in response to mucosal stimuli, with a more exaggerated gd-IgA1 response in those with IgA nephropathy.^8^ COVID-19 vaccination is, however, a systemic vaccination, and thus the precise mechanisms by which it may induce a flare of IgA nephropathy are unclear. One possibility is that the spike protein for the severe acute respiratory syndrome–related coronavirus 2 virus encoded for by the mRNA vaccines may enter respiratory and intestinal mucosal epithelia and provide the mucosal antigenic stimuli required to initiate gd-IgA1 production, which eventually leads to the mesangial inflammation characteristic of IgA nephropathy.^9^ Intriguingly, Aoki *et al.* found no difference in serum gd-IgA1 levels at the time of visible hematuria compared with that 6 months later (it is unclear whether these levels are higher than pre-vaccine baseline levels) but did find urinary gd-IgA1 levels to be elevated at the time of visible hematuria. These findings could be consistent with a global exacerbation of kidney inflammation in the context of pre-existing IgA nephropathy, driven by increased filtration of gd-IgA1 (yielding normal serum levels of gd-IgA1 but elevated urinary levels), accounting for the lower rates of lesions, such as E1, which may indicate the occurrence of more focal inflammation.

Although Aoki *et al.* highlight a risk of IgA nephropathy flares after vaccination against COVID-19, it must also be noted that COVID-19 itself can induce flares of IgA nephropathy. While Aoki *et al.* did not report on a second control cohort of patients who developed visible hematuria after infection with severe acute respiratory syndrome related coronavirus 2, a large prospective observational study of over 2000 patients with glomerular disease from Europe and North America, of which approximately 30% had IgA nephropathy, found that those with COVID-19 suffered from a greater reduction in eGFR slopes compared with those who were vaccinated against COVID-19 (although these figures were not compared directly by statistical testing). Nevertheless, in both groups eGFR was found to be stable at the time of infection/vaccination versus 6 months later. Proteinuria surges were also found to be more severe among those with COVID-19 compared with those who had received a COVID-19 vaccine.^10^

The research conducted by Aoki *et al.* offers further insights into the relationship between COVID-19 vaccination and IgA nephropathy. Their findings suggest that, in their Japanese cohort, the vaccine predominantly triggered flares of preexisting IgA nephropathy rather than inducing new cases. Importantly, these flares appear to be transient and do not affect kidney function in the short term, and this finding is consistent with larger studies performed in other international cohorts. Although further data are needed to fully understand the implications of vaccination in the context of IgA nephropathy, the wider literature would indicate the flares of IgA nephropathy induced by the vaccination may be milder than those induced by COVID-19 itself. In the context of the wider irrefutable protective effects of the COVID-19 vaccines, this should reassure those with IgA nephropathy and the clinicians who care for them of the utility of vaccination.
